# Determine the factors that affected COVID-19 prevention behaviors based on constructs of social cognition theory

**DOI:** 10.1186/s12889-023-17209-y

**Published:** 2023-11-22

**Authors:** Raheleh Soltani, Mohsen Shamsi, Atefe Moradi

**Affiliations:** Department of Health Education and Health Promotion, School of Health, Arak University of Medical Sciences, Arak, Iran

**Keywords:** COVID-19, Prevention and control, Social cognition theory, Self-regulation

## Abstract

**Background:**

COVID-19 is a universal challenge and novel disease is one of the core public health concerns. This study aimed to determine the factors that affected COVID-19 prevention behaviors (CPB) based on constructs of Social Cognition Theory (SCT).

**Methods:**

This cross-sectional study was conducted on 426 participants referred to health centers of Arak from October 2021 to February 2022, and they were selected through a multi-stage random sampling method. Data were collected via a self-administered questionnaire which includes socio-demographic data (6 items), COVID-19 prevention behaviors (12 items), and SCT constructs related to COVID-19 (32 items). SPSS Ver-16 statistical software was used to analyze the data with one-way ANOVA, independent samples *t*-test, and multiple linear regressions. The significance level of α = 0.05 was considered for all tests.

**Results:**

The mean age was 37.8 years (SD = 12.2) and ranged from 17 to 81. According to the results, 51.2% had higher education and 52.6% of the participants were female. The mean (SD) of COVID-19 preventive behaviors was 43 (SD = 7.8) out of 60. There was statistical association between CPB and three constructs of SCT. Multiple regression showed that the outcomes expectations (β = 0.11, *p* < 0.001), self-regulation (β = 0.41, *p* < 0.001), and self-efficacy (β = 0.30, *p* < 0.001), age, gender, and history of COVID 19infection were associated with CPB and those described 61% of the variance of CPB.

**Conclusion:**

Rendering to the result of this study constructs of SCT is the key predictor of participants’ CPB. Hence, based on these predictors, effective interventions and healthy messages could be designed based on this predictor—outcomes expectations, self-regulation, social support, and self-efficacy which can be beneficial to improve healthy behavior.

## Introduction

A new contagious disease was reported in December 2019 and the World Health Organization (WHO) called it COVID-19 on 30 January 2020 [1]. COVID-19 infection, as a universal challenge and novel disease, is one of the core public health concerns [2]. In the last report of WHO on 11 May 2023, globally about 2.7 million new cases of COVID-19 and over 17,000 deaths were reported in 28-day intervals from 10 April to 7 May 2023. Globally over 765 million suffered from COVID-19 and over 6.9 million deaths have been reported. At the six regionals’ WHO, 40,000 new cases were reported in the Eastern Mediterranean in the last 28 days in the last report on 11 May 2023. In this report, the highest numbers of new cases (12 023; 14.3 per 100 000) and new deaths (595; 0.7 per 100 000) were reported in Iran [3].

Toward prevention and surveillance of COVID-19 WHO has delivered numerous guidelines about this situation. It spreads generally from person to person via respiratory droplets, sneezing, cough talk, shouting, or singing [4]. Literature and documents are recommended vaccination and guidelines for COVID-19 prevention behaviors [1, 5, 6]. Despite the recommendation for COVID-19 prevention behaviors, it is inadequate [7, 8] and some studies showed that individuals are against COVID-19 prevention behaviors [9, 10].

Sociologists and psychologists have recommended the use of theories and models to explain the factors affecting health behaviors. Behavioral theories help to better understand the complexity of the factors affecting health behavior and reinforcing educational interventions [11, 12]. Studies designed based on a model or theory can better measure the factors affecting the behavior and determine the reasons for the success or failure of health-related behaviors [13, 14]. It is better to use behavioral theories when you need to know why persons do not follow COVID-19 preventive behaviors and the impact of various factors on the acceptance of behavior [15, 16]. A conceptual review study (2022) on social cognition theories and behavior change in COVID-19″- suggests that apply SCT to predict the controlling of COVID-19 as an endemic disease, booster vaccination. This study also suggested conducting more research using the SCT to determine the main factor affecting the preventive behavior of COVID-19 [17]. The literature recommends that the SCT can be applied to understand adherence to COVID-19 prevention practices [8, 16–18]. Social cognitive theory in 1963 by Bandura was introduced. According to this theory, behavior is described through three factors including individual, behavioral, and environmental or interpersonal social factors. The core elements and key SCT constructs of including outcome expectations, self-regulation, self-efficacy, and social support [19].

We will not need a theory if we know the rate of COVID-19 or the rate of adherence to COVID-19 prevention behaviors. However, when we want to know the impact of various factors on the adoption of healthy behavior, such as why people do not wear masks or why they do not follow COVID-19 prevention behaviors, we will need to use behavioral theory to better explain and understand it.With considering the difference in numerous reports as well as the less use of social cognitive theory studies and the need to investigate the behavior of the people of a community regarding infectious respiratory diseases in the event of outbreaks similar to the Covid-19 disease in the post-corona era were pointed out as existing information gaps in introduction section. Therefore this study aimed to determine the factors affected by COVID-19 prevention behaviors (CPB) based on constructs of the SCT.

## Method

### Study design and population

This cross-sectional study was directed at 426 participants who were referred to the health centers of Arak, from August 2021 to November 2022. Arak is located in central Iran and the center of Markazi province a population of around 600,000.

### Sample size estimation

The sample size was considered 430 based on a 40.9% prevalence of COVID-19 prevention behaviors established in a previous study, with a level of confidence (zα/2 = 1.96), 5% level of precision, and non-response rate (estimated to be 20%). Using the following formula:

$$n=\frac{{Z}^{2}\times p\left(1-p\right)}{{d}^{2}}=426$$

The multi-stage stratified method was used for sample selection. Two health centers were randomly selected from five regions according to socioeconomic status. Then, at each health center eligible participants were randomly selected finally, 426 eligible participants were selected from 10 health centers from five districts of Arak.

The inclusion criteria were willingness to participate in the study and not suffering from special mental and emotional diseases.

### Measuring tools

Data were collected by a self-administered questionnaire which includes socio-demographic data (6 items), COVID-19 prevention behaviors (12 items), and SCT constructs related to COVID-19 (32 items). Data were collected through self-administered and completing the questionnaire took nearly 20 min. The strengthening of the reporting of observational studies in epidemiology (STROBE) statement was applied in this study.

#### Socio-demographic data

Socio-demographic data comprised gender, age, education level (primary, secondary, high school, diploma, and academic), occupation and marital status, and economic status (poor, average, good).

#### COVID-19 prevention behaviors

The COVID-19 prevention behaviors were measured with 12 items. For example, the frequency of mask use was asked and the given score ranged from 1 (never) to 5 (always).

### SCT constructs related to COVID-19

The SCT constructs related to COVID-19 were assessed through 32 items derived from the available literature according to the SCT procedures and guidelines [12, 19]. The SCT constructs include participants’ outcomes expectations (14 items), social support (7 items), self-regulation (6 items), and self-efficacy (5 items) constructs showed in Table 1.

**Table 1:** The constructs of SCT, number of items, range score, validity and reliability

TPB constructs Variables	Items example	Options	Number of items	Range score	Cranach’s Alpha	CVI
Outcomes expectations	Wearing a face mask makes me feel safety	1 = Completely disagree 2 = Disagree 3 = No idea 4 = Agree 5 = Completely agree	14	14–70	0.85	0.81
behavior	After entering the home, I wash my hands with soap and water	1 = never 2 = rarely 3 = sometimes 4 = often 5 = always	12	12–60	0.824	0.79
Social support	My family members encourage me to wear a face mask	1 = never 2 = rarely 3 = sometimes 4 = often 5 = always	7	7–35	0.812	0.76
Self-Regulation	I have planned to follow the preventive behaviors against corona virus	1 = never 2 = rarely 3 = sometimes 4 = often 5 = always	6	6–30	0.78	0.82
Self-efficacy	How confident are you to wear a face mask from leaving home until returning?	1 = not at all confident, 10 = very confident	5	5–50	0.88	0.89

### Validity and reliability

The content validity of measuring tools was established by the expert panel of ten academicians in this field (health education and health promotion experts and health care providers). The content validity ratio (CVR), content validity index (CVI), and reliability (Cronbach’s alpha for each item are presented in Table 1. The test–retest factor was 0.82 (2 weeks interval and among 25 participants).

### Ethical considerations

The Ethical Committee of the Arak University of Medical Science, Iran, approved the study protocol (ID number- IR.ARAKMU.REC.1399.312). The purpose of the study was explained to the participants, and written consent was then obtained from participating mothers who volunteered to enter this study. Informed consent from illiterate participants were completed through face-to-face interviews at each health centre.

### Statistical analysis

Data were analyzed through SPSS version 16 (SPSS, Inc., Chicago, IL, USA) statistical software using one-way ANOVA (with Bonferroni post hoc method), independent samples *t*-test, and multiple linear regressions. The significance level of *α* was considered 0.05 for all data. Data were analyzed by SPSS 16 using.

## Results

The mean age was 37.8 years (SD = 12.2)and ranged from 17 to 81. Results showed that 52.6% (224/426) of the participants were female and 51.2% (281/426) had higher education. The mean (SD) of COVID-19 preventive behaviors was 43 (SD = 7.8) out of 60. About 241 participants (56.6%) had adequate self-regulation (95% Confidence Interval [CI] = 49.1%-70.6%), participants’ adequate self-efficacy was 60.3% (257/426) (95% CI = 39. 9% ‒79.6%), and 394 participants (92.5%) had intermediate outcomes expectations (95% CI = 63. 8% ‒98. 8%). Table 2 displays the association of SCT constructs related to COVID-19 and socio-demographic characteristics with the mean score of CPB. Economic status, education, and gender (*p* < 0.05) were statistically associated with CPB. Moreover according to the post hoc Bonferroni test, the average of preventive behaviors of Covid-19 is higher in people with over than 75 years old, people with higher socio-economic statuses and higher education, and people with higher self-regulation (*p* < 0.001).

**Table 2:** Distribution of socio-demographic characteristics, Mean and SD of CPB and their associated factors (*n* = 426)

Variables	Category	N (%)	CPB Mean (SD)	*P*-value	(df) f
Age	< 25	79 (18.5%)	39.9 (7.5)	0.001	(2,423)7.5
	25–50	293 (68.8%)	43.7 (7.9)		
	50–75	54 (12.7%)	43.8 (6.7)		
Gender	Female	224 (52.6%)	44.8 (7.3)	< 0.001	
	Male	202 (47.4%)	41 (7.8)		
History of covid 19 infection	Yes	226 (53.1%)	42.7 (7.4)	0.274	
	No	200 (46.9)	43.3 (8.2)		
Marital status	Single	139 (32.6%)	40.7 (8.1)	< 0.001	
	Married	287 (67.4%)	44.1 (7.4)		
Vaccine	Yes	384 (90.1%)	43.3 (7.7)	0.006	
	no	42 (9.9%)	39.6 (8.4)		
Economic Status	Low	75 (17.6%)	39.7 (7.8)	< 0.001	(2,423)10.1
	Medium	194 (45.5%)	43 (7.4)		
	High	157 (36.9%)	44.5 (7.9)		
Educational level	Illiterate and Elementary	25 (5.9%)	38.6 (7.2)	0.014	(3,422)3.5
	High school graduate	47 (11%)	42.3 (7.1)		
	diploma	136 (31.9%)	42.8 (7.9)		
	College education	218 (51.2%)	43.8 (7.8)		
Outcomes expectations	Low	32 (7.5%)	34.3 (6.1)	< 0.001	
	Medium	394 (92.5%)	43.7 (7.5)		
	High	0	0		
Behaviors	Low	0	0	< 0.001	
	Medium	159 (37.3%)	34.6 (4)		
	High	267 (62.7%)	48 (4.7)		
Social support	Low	6 (1.4%)	45.1 (10.4)	0.608	
	Medium	420 (98.6%)	42.9 (7.8)		
	High	0	0		
Self-Regulation	Low	12 (2.8%)	30.2 (3.7)	< 0.001	(2,423)140
	Medium	173 (40.6%)	38 (6)		
	High	241 (56.6%)	47.2 (6.2)		
Self-efficacy	Low	20 (4.7%)	32.2 (6.2)	< 0.001	(2,423)112
	Medium	149 (35%)	38.2 (6.6)		
	High	257 (60.3%)	46.6 (6.2)		

There was a statistical association between CPB and three constructs of SCT (outcomes expectations, self-regulation, and self-efficacy) (Table 3). Multiple regression showed that outcomes expectations (β = 0.11, *p* < 0.001), self-regulation (β = 0.41, *p *< 0.001), and self-efficacy (β = 0.30, *p* < 0.001), age, gender, and history of COVID 19infection were associated with CPB and those described 61% of the variance of CPB (Table 4). The distribution of each COVID-19 preventive behavior of the study participants is shown in Table 5 the rate of “Always” and “often” washing hands was 61.3% and 20.9% respectively.

**Table 3:** Correlation coefficients between each of the continuous variables

**Variable**	1 r(p)	2r(p)	3 r(p)	4 r(p)	Mean (SD)
1 = Outcomes expectations	1				30.3 (4.8)
2 = Self-efficacy	0.55(0.001)	1			35.(9.2)
3 = Self-Regulation	0.66 (0.001)	0.66(0.001)	1		28.6(6.3)
4 = Social support	0.43(0.001)	0.33(0.001)	0.53(0.001)	1	25.4(6)
5 = Behavior	0.59(0.001)	0.65(0.001)	0.72(0.001)	0.38(0.001)	43 (7.8)

**Table 4:** Regression analysis of predictor factors of behavior

Variable	Unstandardized coefficients		*t*	*P*	95%Confidence Interval for B		Collinearity Statistics		Adjusted *R*^2^
	*B*	SE			Lower Bound	Upper Bound	Tolerance	VIF	
Self-Regulation	0.51	.063	8.2	< 0.001	0.39	0.64	0.35	2.8	0.61
Self-efficacy	0.25	0.03	7.0	< 0.001	0.18	0.32	0.49	2	
Outcomes expectations	0.18	0.07	2.6	0.008	0.05	0.32	0.48	2	
Age	0.04	0.02	2.0	0.036	0.0	0.08	0.79	1.2	
Gender	1.6	0.5	3.3	0.001	2.6	0.68	0.86	1.1	

*R* = 0.788, f(df) = 85.3(8,417), *P* < 0.001, F Change = 85.3, *R* Square Change = 0.62, *P* < 0.001

**Table 5:** Distribution of each COVID**-**19 preventive behaviors of the study participants

	Never N (%)	Rarely N (%)	Sometimes N (%)	Often N (%)	Always N (%)
Outdoor face mask use	5 (1.2%)	13 (3.1%)	43 (10.1%)	106 (25.6%)	256 (60.1%)
Wear a face mask during a family gathering	160 (37.6%)	106 (24.9%)	73 (17.1%)	67 (15.7%)	20 (4.7%)
Avoiding any mass family gatherings	77 (18.1%)	95 (22.3%)	137 (32.2%)	88 (20.7%)	29 (6.8%)
Washing hands and face after coming from outside	5 (1.2%)	17 (4%)	54 (12.7%)	89 (20.9%)	261 (61.3%)
Maintaining physical distance	13 (3.1%)	69 (16.2%)	126 (29.6%)	152 (35.7%)	66 (15.55)
Aoid of crowded areas and social events	19 (4.5%)	68 (16%)	117 (27.5%)	131 (30.8%)	91 (21.4%)
leave home only for vital daily activities	42 (9.9%)	96 (22.5%)	96 (22.5%)	100 (23.5%)	92 (21.6%)
Covering mouth/nose while coughing or sneezing	1 (0.2%)	13 (3.1%)	30 (7%)	86 (20.2%)	296 (69.5%)
Avoiding unnecessary travel	39 (9.2%)	42 (9.9%)	105 (24.6%)	123 (28.9%)	117 (27.5%)
Using the hand sanitizer outdoor	71 (16.7%)	63 (14.8%)	79 (18.5%)	82 (19.2%)	131 (30.8%)
Cash payment in shopping	18 (4.2%)	36 (8.5%)	104 (24.4%)	141 (33.1%)	127 (29.8%)
Dining inside restaurants and public places	11 (2.6%)	33 (7.7%)	98 (23%)	142 (33.3%)	142 (33.3%)

## Discussion

This study discovered the association between SCT constructs and COVID-19 preventive behaviors (CPB). To our knowledge, few documents have been published about the CPB via STC constructs.

In the present study, the self-regulatory (SR) structure influenced the participant’s CPB and the mean score of CPB among participants with adequate SR status was significantly higher than that of poor SR (47.2 vs 30.2). Lin et al.’s study [20], aimed to predict preventive behaviors of COVID-19 based on the SCT on 1718 participants, showed that action planning was one of the predictive structures of CPB. Also, this finding confirms the study of Sousa et al. [21] In this study it was also observed that the self-regulation structure directly affects health-related habits and mental health during the COVID-19 pandemic. Self-regulation is the process of setting goals and planning to perform the desired behavior by the individual [22] and the improvement of this structure in the individual leads to the facilitation of OVID-19 preventive behaviors. This structure can help a person to develop and implement his planned behavior and maintain it flexibly until he reaches his desired final goal [23, 24]. People with higher self-regulation maintain their behavior over time despite failures and adversities [25].

In a study Lauren et al. [26] about barriers to COVID-19 prevention behaviors among North Carolina residents showed that the most commonly endorsed barriers to 6-foot distancing included physical impediments, forgetting, and unfavorable descriptive norms.

Another result of this study was the positive effect of outcome expectations on adopting CPB. It can be said individuals who had an intermediate level of outcome expectations had a higher score CPB compared to people who had limited outcome expectations (43.7 vs. 34.3), which confirms the previous studies by Cavicchiolo et al. [27] and Sinicrope et al. [28].

In a study by Coroiu et al. [29] about barriers and facilitators of adherence to social distancing recommendations during COVID-19 among a large international sample of adults indicated that adherence to social distancing recommendations vary depending on the behaviour, with none of the surveyed behaviors showing perfect adherence. Strongest facilitators included wanting to protect the self, feeling a responsibility to protect the community, and being able to work/study remotely; strongest barriers included having friends or family who needed help with running errands and socializing in order to avoid feeling lonely.

A study by sinicrope et al. showed that the perceived outcomes expectations toward using a mask are effective on the behavior of using a mask [28]. Also, this finding is similar to a study that aimed to investigate behavioral intention to inject COVID-19 vaccination based on social cognitive theory, which showed that outcomes expectations of physical and self-evaluative outcomes were related to behavioral intention of COVID-19 vaccination among doctors and nurses [5].

In a study about COVID-19 vaccine acceptance and hesitancy in low- and middle-income countries indicated that messages highlighting vaccine efficacy and safety, delivered by healthcare workers, could be effective for addressing any remaining hesitancy in community [30]. Hence, health promotion can highlight the benefits of COVID-19 prevention behaviors for both individuals and communities.

This study displays that COVID-19 prevention behaviors were significantly and positively associated with participants’ self-efficacy towards COVID-19 prevention behaviors (*R* = 0.55, *P* < 0.001). This result is reliable with previous studies that found a significant association between self-efficacy and COVID-19 prevention behaviors [29, 31]. In a study by Soltani et al. self-efficacy was significant a predictor of mask-wearing habits during the COVID-19 pandemic [15]. Barrett et al. also found self-efficacy associated with social distancing behavior during the COVID-19 pandemic among university students in the UK [31]. Self-efficacy is one of the key requirements to develop and keep health-related behavior [19]. Therefore, improving self-efficacy can lead to health habits and promotion of self-care behavior. Hence, based on improving self-efficacy through educational interventions and community massage could be useful.

Social support is the support that a person receives from others, especially family members and friends, to perform the desired behavior [32, 33] and is one of the key constructs of the SCT. In fact family is a crucial environmental factor influencing people’s health attitudes and behaviors.

Wanqi et al. [34] in a study about the role of family communication patterns in intergenerational COVID-19 discussions and preventive behaviors showed that the family communication patterns can be associated with preventive behaviors through different forms of family discussion about COVID-19. Conversation orientation is a strong facilitator for positive behavioral effects and scientific discussion is the most benign form of family health discussion. Health communication efforts should enhance the agency role of the family and motivate scientific discussion in health practices.

In the present study, there was a weak correlation between social support and CPB, which was not significant in the regression analysis. Kowalsky et al.’ study during the COVID-19 pandemic based on the social cognitive model, indicated a weak correlation was seen between subjective norms and behavior [33].

This study exposed that SCT theory was a significant predictor of the CPB and it described 61% of the variance. We found the clear effects of STC constructs (self-regulation, self-efficacy, and outcome expectancy) on participants’ COVID-19 preventive behaviors. These results are consistent with some studies. In the study by Raude et al. [18] which was conducted to investigate COVID-19 preventive behaviors based on cognitive, psychological, cultural, and social variables in France, cognitive variables predicted 23% of CPB. A study by Cavicchiolo et al., on 363 teenagers in 5 schools in Italy, demonstrated the structure SCT predicted 25% of the social distancing behavior during the COVID pandemic [27]. An online cross-section study by Barrett et al. shows that social cognitive model predicted 40% of hand washing and 25% of social distancing behavior [31]. In primary health care settings, this matter is vital for health services that design appropriate educational intervention programs based on SCT constructs about healthy behavior such as CPB.

In this study the almost participant use outdoor face mask, maintaining physical distance and avoid of crowded areas and social events. Also, this finding is similar to other studies [7, 10, 29].

There were some limitations in this study. First, the questionnaire was self-reported which could be subject to recall and response bias. Second, this study, might not lead to the casual interpretations of constructs of SCT and CPB. Despite limitations, the seemly, valid, and cost-effective method of data collection is self-assessment on adolescents and adults. This study also can create some other scientific evidence for future studies about health promotion communities.

## Conclusion

Rendering to the result of this study constructs of SCT is the key predictor of participants’ CPB. Hence to design useful and effective behavioral change interventions based on evidence in the field of COVID-19 prevention. Finally based on these predictors, effective interventions and healthy messages could be designed based on this predictor outcomes expectations, self-regulation, social support, and self-efficacy which can be beneficial to improve healthy behavior.

## Data Availability

The datasets analyzed during the current study are available from the corresponding author on reasonable request.

## Abbreviations

SCT: Social Cognitive Theory
CVR: Content Validity Ratio
CVI: Content Validity Index
SD: Standard Deviation
